# An American Cereal—Cornmeal

**Published:** 1917-06

**Authors:** 


### An American Cereal—Cornmeal.

Commenting on the increase in the price of bread announced by the large bakers, the Department of Health has again directed attention to cornmeal—America's food for everybody.

Cornmeal contains exactly the same amount of nourishment as wheat flour and is more than 25 per cent cheaper. It is even cheaper than rice.

One pound of wheat flour contains 1675 food units, and costs 8 cents.

One pound of rice (broken) contains 1600 food units, and costs 7 cents.

One pound of cornmeal contains 1680 food units and costs 5 cents.

Cornmeal may be used for a large variety of dishes. In the form of mush, it is an excellent breakfast cereal; fried, it is a pleasing and satisfactory substitute for potatoes.

A dish of fried cornmeal mush for four persons costs 5 cents. If slices of mush are browned in a pan and served with minced cheese and a little butter, a tempting dish for four persons can be provided for 15 cents.

### EVER EAT POLENTA?

In England the most common substitute for potatoes is an Italian dish known as "polenta," a kind of porridge made of corn instead of oats. Unlike other Italian dishes, it does not require an expert to cook it, and the flour can be bought today at 5 cents the pound. Half a pound suffices for a meal for five people, this costing less than the potatoes necessary for an equal number and being much more substantial. To the Italian, polenta is what porridge is to the Scotsman. In London, at the present time, macaroni has become so popular that it is now scarce and dear. We shall probably witness the same thing here. The next best the Italian cook has to offer is polenta. It will be worth trying.

